# Effects of retinoic acid receptor α modulators on developmental ethanol-induced neurodegeneration and neuroinflammation

**DOI:** 10.3389/fnins.2023.1170259

**Published:** 2023-05-02

**Authors:** Mariko Saito, Shivakumar Subbanna, Xiuli Zhang, Stefanie Canals-Baker, John F. Smiley, Donald A. Wilson, Bhaskar C. Das

**Affiliations:** ^1^Division of Neurochemistry, Nathan Kline Institute for Psychiatric Research, Orangeburg, NY, United States; ^2^Department of Psychiatry, New York University School of Medicine, New York, NY, United States; ^3^Emotional Brain Institute, Nathan Kline Institute for Psychiatric Research, Orangeburg, NY, United States; ^4^Department of Child and Adolescent Psychiatry, New York University Medical Center, New York, NY, United States; ^5^Arnold and Marie Schwartz College of Pharmacy and Health Sciences, Long Island University, Brooklyn, NY, United States; ^6^Department of Medicine, Icahn School of Medicine at Mount Sinai, New York, NY, United States

**Keywords:** fetal alcohol spectrum disorders, astrocyte, GABAergic neuron, boron-containing small molecules, retinoic acid receptor **α** agonist, retinoic acid receptor **α** antagonist, neurodegeneration, neuroinflammation

## Abstract

Ethanol exposure in neonatal mice induces acute neurodegeneration followed by long-lasting glial activation and GABAergic cell deficits along with behavioral abnormalities, providing a third trimester model of fetal alcohol spectrum disorders (FASD). Retinoic acid (RA), the active form of vitamin A, regulates transcription of RA-responsive genes and plays essential roles in the development of embryos and their CNS. Ethanol has been shown to disturb RA metabolism and signaling in the developing brain, which may be a cause of ethanol toxicity leading to FASD. Using an agonist and an antagonist specific to RA receptor α (RARα), we studied how RA/RARα signaling affects acute and long-lasting neurodegeneration and activation of phagocytic cells and astrocytes caused by ethanol administered to neonatal mice. We found that an RARα antagonist (BT382) administered 30 min before ethanol injection into postnatal day 7 (P7) mice partially blocked acute neurodegeneration as well as elevation of CD68-positive phagocytic cells in the same brain area. While an RARα agonist (BT75) did not affect acute neurodegeneration, BT75 given either before or after ethanol administration ameliorated long-lasting astrocyte activation and GABAergic cell deficits in certain brain regions. Our studies using Nkx2.1-Cre;Ai9 mice, in which major GABAergic neurons and their progenitors in the cortex and the hippocampus are labeled with constitutively expressed tdTomato fluorescent protein, indicate that the long-lasting GABAergic cell deficits are mainly caused by P7 ethanol-induced initial neurodegeneration. However, the partial reduction of prolonged GABAergic cell deficits and glial activation by post-ethanol BT75 treatment suggests that, in addition to the initial cell death, there may be delayed cell death or disturbed development of GABAergic cells, which is partially rescued by BT75. Since RARα agonists including BT75 have been shown to exert anti-inflammatory effects, BT75 may rescue GABAergic cell deficits by reducing glial activation/neuroinflammation.

## Introduction

Prenatal alcohol exposure causes adverse effects on fetal development especially in the brain, leading to fetal alcohol spectrum disorders (FASDs) in offspring. FASD neuropathology includes volume reduction in the corpus callosum, cerebral cortex, cerebellum, and subcortical structures such as the hippocampus, basal ganglia, amygdala, and thalamus, which associate with cognitive and behavioral abnormalities (Riley and McGee, 2005; Astley et al., 2009; May et al., 2009; Norman et al., 2009; Riley et al., 2011; May et al., 2014). While many factors can be involved in pathogenesis of FASD, retinoic acid (RA) signaling has been considered an important factor, based on clinical studies as well as studies using animal models for FASD (Duester, 1991; Johnson et al., 2007; Sapin, 2008; Kane et al., 2010; Young et al., 2014; Fainsod et al., 2020). RA, the active form of vitamin A, regulates transcription of RA-responsive genes *via* nuclear RA receptor (RAR)-α,β,ɣ and retinoid X receptor (RXR)-α,β,ɣ activation along with less frequent non-genomic regulation and plays essential roles in development of embryos and their CNS as well as in adult tissue homeostasis (Mey and Mccaffery, 2004; Niederreither and Dollé, 2008; Crandall et al., 2011; Rhinn and Dollé, 2012; Shearer et al., 2012; Haushalter et al., 2017; Dowling, 2020). Temporal and spatial RA distribution within embryonic cell population seems to be precisely controlled by dynamic expression patterns of synthesizing/metabolizing enzymes (Niederreither and Dollé, 2008; Rhinn and Dollé, 2012), and insufficient or excess RA causes teratogenic and/or toxic effects, which mimic the ethanol effects on the developing brain (Wolf, 2010; Fainsod et al., 2020). It has been shown that ethanol reduces RA levels due to the competition between ethanol clearance and RA biosynthesis (Duester, 1991; Johnson et al., 2007; Fainsod et al., 2020). The reduction in RA induces multiple deficits similar to FASD in both patients and animal models, and addition of RA or RA synthesizing enzymes restores the deficits in adult or embryonic brains of several species (Yelin et al., 2005; Johnson et al., 2007; Marrs et al., 2010; Shabtai et al., 2018). On the other hand, several reports indicate that both acute and chronic alcohol elevate RA in adult and fetal brain (Kane et al., 2010) and in the developing or adult cerebellum (McCaffery et al., 2004), and an antagonist of RARβ suppresses RARβ expression and reverses chronic ethanol-induced cognitive impairment (Alfos et al., 2001). The discrepancy may be due to the differences in the age and alcohol administration conditions.

Ethanol exposure in neonatal rodents causes acute neurodegeneration as well as long-lasting neuroanatomical and behavioral deficits, providing a third trimester binge drinking model. Previous studies have demonstrated that ethanol exposure in the P7 mouse brain induces robust acute neurodegeneration within 24 h in many brain regions especially in the cingulate and retrosplenial cortex and the thalamus (Olney et al., 2002a; Saito et al., 2007). These brain regions belong to the extended hippocampus circuitry important for spatial learning and memory functions, which may explain profound learning and memory deficits observed in this mouse model of FASD (Wozniak et al., 2004). The acute neurodegeneration detected by cleaved caspase 3 expression, Fluoro-Jade (FJ) C, and Tunel assay is accompanied by activation of microglia, which phagocytose FJ positive degenerating neurons, the subsequent activation of astrocytes, and long-lasting GABAergic cell deficits (Coleman et al., 2012; Saito et al., 2015; Smiley et al., 2015, 2019; Bird et al., 2018, 2020; Saito et al., 2019). The GABAergic cell deficits observed mainly in parvalbumin (PV) positive (+) and somatostatin (SST) + cells are more prominent than the total neuron deficits and may cause neurobehavioral abnormalities (Sadrian et al., 2012, 2014; Wilson et al., 2016; Lewin et al., 2018). However, mechanisms of this relatively specific long-lasting GABAergic cell loss are unclear. GABAergic cells or their progenitors exposed to P7 ethanol may die more than other types of neurons during acute neurodegeneration, and/or may be specifically affected by delayed cell loss or perturbed PV/SST expression. Since disturbed RA signaling is involved in ethanol toxicity in the developing brain (Duester, 1991; Alfos et al., 2001; McCaffery et al., 2004; Yelin et al., 2005; Johnson et al., 2007; Sapin, 2008; Kane et al., 2010; Marrs et al., 2010; Young et al., 2014; Shabtai et al., 2018; Fainsod et al., 2020) and influences GABAergic cell maturation in early postnatal mouse prefrontal cortex (Larsen et al., 2019), we examined how RARα modulation affects the course of P7 ethanol-induced neurodegeneration and neuroinflammation using a specific RARα agonist BT75 and an antagonist BT382. BT75 was previously synthesized as a borate containing derivative of RARα agonist Am580 and has been shown to be less toxic compared to all-trans retinoic acid (ATRA) or Am580 (Zhong et al., 2011). BT382 is an RARα antagonists identified in our previous studies (Anguiano et al., 2013).

Our present studies indicated that while BT382 partially attenuated P7 ethanol-induced acute neurodegeneration, BT75 alleviated long-term astrocyte activation and GABAergic cell loss in the cortex and hippocampus. RA signaling activation by BT75 may attenuate chronic neuroinflammation and improve survival/maturation of GABAergic neurons in the adult brain. Thus, our studies suggest that RA signaling is a promising target for developing neuroprotective agents against FASD.

## Materials and methods

### Syntheses of BT382 and BT75

**Figure 1:**
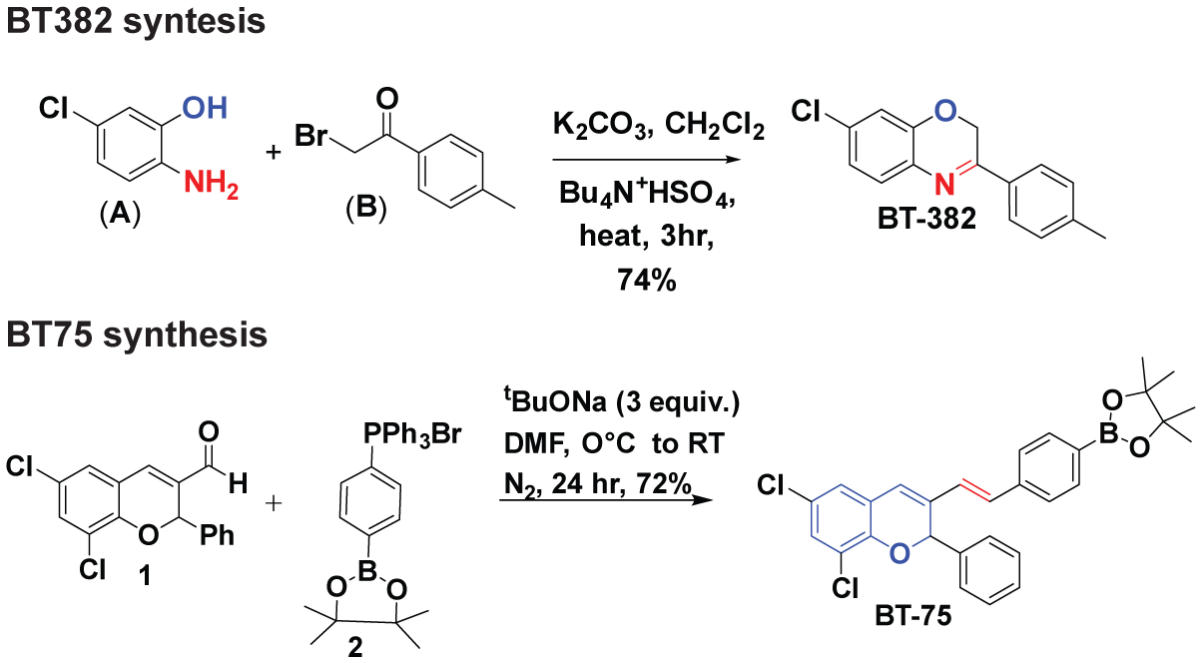
Synthesis of BT382 and BT75.

Synthesis of BT382 (Figure 1) was performed as follows: To a solution of 2-amino-5-chlorophenol (A) (0.001 mol) in dichloromethane (40 ml) aqueous potassium carbonate (20% w/v) and tetrabutylammonium hydrogen sulfate (0.0005 mol) was added and mixture was stirred for 2 h at room temperature. After 2 h, 2-Bromo-4-methylacetophenone (0.01 mol) in 20 ml dichloromethane was added drop-wise through a course of 15 min and the resultant mixture was refluxed till completion for 3 h. The organic layer was extracted with dichloromethane and dried over sodium sulfate evaporated in vacuum to give crude solid product. The solid was then recrystallized with hot ethanol to obtain pure BT382. The purity was verified using NMR (1H, 13C), HRMS, and HPLC. The result of BT382 [7-Chloro-3-(p-tolyl)-2H-benzo(b)(1,4)oxazine]: (Yield = 77%), 1H NMR (600 MHz, DMSO-d6) δ 7.89 (d, *J* = 8.0 Hz, 2H), 7.33–7.31 (m, 3H), 7.08–7.02 (m, 2H), 5.19 (s, 2H), 2.48 (s, 3H). HRMS (ESI) calcd for C15H13ClNO 258.0680 found 258.1377 [M + H] + .

Synthesis of BT75 [Figure 1; (E)-2-(4-(2-(6,8-dichloro-2-phenyl-2H-chromen-3-yl)vinyl)phenyl)-4,4,5,5-tetramethyl-1,3,2-dioxaborolane] was performed as follows: A clean oven dried three-neck round bottom flask (RBF) was charged with aldehyde 1 (Das et al., 2010) (1 equiv.) in DMF. Then addition of salt 2 (1.2 equiv.) Sodium tertbutoxide (STB) (3 equiv.) was added into the reaction mixture at 0°C. The reaction mixture was stirred at room temperature for 24 h. Progress of the reaction was monitored by TLC (100% Hexane). In TLC nonpolar spot was observed corresponding to aldehyde. After completion of the reaction, reaction mixture was neutralized by 2 N HCl. After neutralization, ethyl acetate was added in the reaction mixture; the combined organic layer was collected and dried with Na_2_SO_4_ and evaporated under reduced pressure. The crude material was purified by column chromatography (5% ethyl acetate: 95% hexane). After purification white solid was observed (BT75). The result of BT75 synthesis was: (Yield = 72%), ^1^H NMR (600 MHz, DMSO-*d*_6_) δ 7.61 (d, *J* = 7.8 Hz, 2H), 7.47 (d, *J* = 7.8 Hz, 2H), 7.42 (d, *J* = 7.2 Hz, 2H), 7.35–7.29 (m, 5H), 7.23 (d, *J* = 16.4 Hz, 1H), 7.01 (s, 1H), 6.71 (d, *J* = 16.5 Hz, 1H), 6.64 (s, 1H), 1.26 (s, 12H).

### Animals

C57BL/6 J (#000664), C57BL/6 J-Tg(Nkx2-1-cre)2Sand/J (Nkx2.1-Cre, #008661), and B6.Cg-Gt(ROSA)26Sortm9(CAG-tdTomato)Hze/J (Ai9, #007909) mice were obtained from Jackson laboratories (Bar Harbor, ME) and bred at the Nathan Kline Institute animal facility under *ad lib* food and water at all times. All procedures were approved by the Nathan Kline Institute IACUC and were in accordance with NIH guidelines for the proper treatment of animals.

### Treatments of C57BL/6 J mice with ethanol, BT75, and BT382

P7 C57BL/6 J pups (both males and females) were injected subcutaneously (sc) with saline or ethanol (2.5 g/kg) twice at a 2 h interval as previously described (Olney et al., 2002a; Saito et al., 2007). Pups were returned to their home cage immediately following injections. Our previous studies showed that this P7 ethanol treatment induced a peak blood alcohol level (BAL) of 0.45 g/dl when truncal blood was collected at 0.5, 1, 3, and 6 h following the second ethanol injection and analyzed with an Alcohol Reagent Set (Pointe Scientific, Canton, MI, United States) (Saito et al., 2007). Pups were weaned at P28 into group cages of littermates. Same-sex mice were housed together in cages in numbers between two and four per cage. For BT75 and BT382 treatments, 2 μl (2.5 or 25 μg in 10% DMSO) of BT75, BT382, or 2 μl of the vehicle (10% DMSO) was administered by intracerebroventricular (icv) injection (Sadakata et al., 2007) 30 min before the first ethanol injection. At P8 (20 h after the first ethanol injection), P60, or P90, mice were perfusion-fixed for immunohistochemistry, or the cortex and hippocampus were dissected out and immediately frozen for Western blot analyzes. For the post-ethanol treatment of BT75, mice were injected intraperitoneally (ip) with BT75 (10 mg/kg, 20 μl/g in 10% DMSO) or vehicle (10% DMSO) once a day for 3 consecutive days starting 3 days after the first ethanol injection at P7. Mice were perfusion-fixed or the cortex and hippocampus were taken out at P30. Since significant sex differences in the effects of P7 ethanol have not been previously observed (Wilson et al., 2016; Saito et al., 2019), nor were any significant differences observed between sexes here, the data from males and females were combined.

### Treatments of Nkx2.1-Cre;Ai9 mice with ethanol

Nkx2.1-Cre mice expressing Cre under the control of the Nkx2.1 promoter/enhancer regions were bred to Ai9 Cre-reporter mice, and the offspring (Nkx2.1-Cre;Ai9) were used for experiments. Since Nkx2.1 is a transcription factor, transiently (around embryonic day 10–14) expressed in the pallidal telencephalon and required for the specification of PV and SST cortical and hippocampal interneurons (Xu et al., 2008), PV and SST neurons and their progenitors in the postnatal cortex and the hippocampus can be identified by a constitutively expressed reporter florescent protein (tdTomato). These mice are useful for studying the fate of PV and SST neurons and their progenitors because of their tdTomato expression in these cells regardless of the presence of PV/SST. To examine the effects of P7 ethanol on acute and long-lasting GABAergic cell loss, Nkx2.1-Cre;Ai9 mice were treated with ethanol at P7 as described above for C57BL/6 J mice and perfusion-fixed at P8, P14, and P30 for histochemical analyzes of tdTomato+ cells. For comparison, immunohistochemical analyzes of PV+ and SST+ cells were also carried out using the same brain sections.

### Fluoro-jade staining, immunohistochemistry and cell counting

In each experiment, 4 to 6 mice per each group derived from at least 3 different litters were used. Mice were perfused with a solution containing 4% paraformaldehyde and 4% sucrose in cacodylate buffer (pH 7.2), and the heads were removed and further fixed in the perfusion solution overnight. Then brains were removed, transferred to phosphate buffered saline (PBS) solution, and kept at 4°C for 2–5 days until cut with a vibratome into 50 μm thick coronal sections. For Fluoro-Jade C (FJ) (Sigma-Aldrich) staining, the free-floating sections rinsed in PBS were placed on slide glasses and processed as described in the manufacturer’s instruction. For immunohistochemistry, the free-floating sections were rinsed in PBS, permeabilized in methanol for 10 min, and incubated for 30 min in blocking solution (PBS containing 5% BSA and 0.1% Triton X-100), followed by incubation overnight with antibodies against parvalbumin (PV) (PV25, Swant, Marly, Switzerland, used for detection of PV neurons), somatostatin (SST) (rabbit polyclonal antibody AP33464SU-N, OriGene, detection of SST neurons), GFAP (D1F4Q) (Rabbit mAb #12389, Cell Signaling, detection of astrocyte activation), GFAP (GA5) (Mouse mAb #3670, Cell Signaling), CD68 (Rat anti mouse monoclonal FA-11, BioRad, detection of phagocytic cell activation), phospho-NF-κB p65 (Ser536) (93H1) (Rabbit mAb #3033, Cell Signaling, detection of NF-κB activation), and NF-κB p65 (D14E12) (Rabbit mAb #8242, Cell Signaling) in PBS containing 3% BSA and 0.1% Triton X-100. Activation of NF-κB, a key nuclear transcription factor, is known to mediate inflammatory responses. Activated NF-κB translocates to the nucleus and is involved in the regulation of transcription of many inflammation-related genes. Furthermore, once activated, posttranslational modifications including phosphorylations allow the regulation of NF-κB transcriptional activity. Especially phosphorylation of p65 (a subfamily of NF-κB proteins) at serine 536 is important for P65 nuclear import (Mattioli et al., 2004). Here, antibodies against phospho (ser536)-NF-κB p65 and NF-κB p65 were used to examine the possibility of glial activation/inflammation through NF-κB activation. Brain sections incubated with one of these primary antibodies were then rinsed in 0.1% Triton X-100 in PBS three times and incubated with another primary antibody for 2 h at r.t., followed by incubation with Alexa Fluoro594 (or 488) goat anti-rabbit (mouse, rat) IgG (Life Technologies, Grand Island, NY) in 0.1% Triton X-100 in PBS containing 1% BSA for 1 h at r.t. Sections were finally rinsed in PBS three times, mounted, and coverslipped using ProLong Gold Antifade Reagent (Life Technologies). All photomicrographs were taken through a 4X, 10X, or 20X objective with a Nikon Eclipse TE2000 inverted microscope attached to a digital camera DXM1200F. Because our previous studies (Smiley et al., 2015) showed that both 2-dimensional and stereological 3-dimensional counting methods gave similar significant reduction in PV cell densities in the cortex by P7 ethanol treatment, the two-dimensional counting method was used in the present studies. The PV, SST, GFAP, and CD68 positive (+) cell number of each area of interest (AOI) and total dimensions of each AOI were measured using the Image-Pro software version 6.0 (Media Cybernetics, Silver Spring, MD). AOIs for cell counting were the cingulate cortex, retrosplenial cortex, dorsal hippocampal molecular layer of dentate gyrus, and dentate gyrus. These AOIs were defined according to the Atlas of mouse brain (Paxinos and Franklin, 2004) or Atlas of the Developing Mouse Brain at E17.5, P0, and P6 (Paxinos et al., 2007). The cell density of each AOI was calculated as the mean cell number per square millimeter from 4 to 6 mice (derived from 3 to 5 different litters) using 4 to 6 sections around bregma 0.98 to 0.14 mm for the cingulate cortex and 3 to 6 sections around bregma -1.22 to -1.94 mm for the retrosplenial cortex/dentate gyrus/molecular layer of dentate gyrus. However, this cell counting method does not provide total cell number in each brain region and is considered a semi-quantitative method, which is a limitation of our current study.

### Brain tissue homogenate and Western blots

Brain tissues (cortex and hippocampus) were homogenized in 10:1 *v/w* tissue homogenization buffer (0.25 M sucrose, 20 mM Tris–HCl pH 7.4, 1 mM EDTA, 1 mM EGTA, all reagents from Sigma-Aldrich) supplemented with protease inhibitors (5 μg/ml leupeptin, 5 μg/ml antipain dihydrochloride, 5 μg/ml pepstatin A, 1 mM phenylmethanesulfonyl fluoride, 1 μM E64, all reagents from Sigma-Aldrich) and phosphatase inhibitors (PhosSTOP, Sigma) immediately before homogenization. The procedure was performed in ice-cold glass homogenizers with 20 complete strokes of Teflon pestles (Wheaton, DWK Life Sciences). The tissue homogenate analysis was performed on equal protein amounts (20 μg), as estimated by the BCA assay (Pierce, Thermo Scientific), and normalized to β-actin. The primary antibodies used are GFAP (D1F4Q) (Rabbit mAb #12389, Cell Signaling), phospho-NF-κB p65 (Ser536) (93H1) (Rabbit mAb #3033, Cell Signaling) and β-Actin (13E5) (Rabbit mAb #4970, Cell Signaling). The secondary antibodies (HRP-conjugated) were from Jackson ImmunoResearch (West Grove, PA, United States). The chemiluminescent substrate was either ECL or femto ECL (both from Pierce, Thermo Fisher Scientific) for either strong or weak signals, respectively. All protein bands were acquired with the iBright FL1500 imaging system (Thermo Fisher Scientific). For densitometry quantification of protein bands, the ImageJ software (National Institute of Health) was used. All histograms were plotted as mean ± standard error of the mean (SEM).

### Statistics

Data were analyzed by Student’s *t*-test for comparisons of two groups and one–or two-way ANOVA for comparisons of more than two groups/factors using SPSS statistics software (version 22). For *post hoc* analyzes, Tukey HSD or Fisher’s LSD groups tests were used. For all analyzes, *p* < 0.05 was considered statistically significant. Values are expressed as mean ± SEM obtained from 4 to 6 animals.

## Results

### BT382, but not BT75, attenuated P7 ethanol-induced acute neurodegeneration

**Figure 2:**
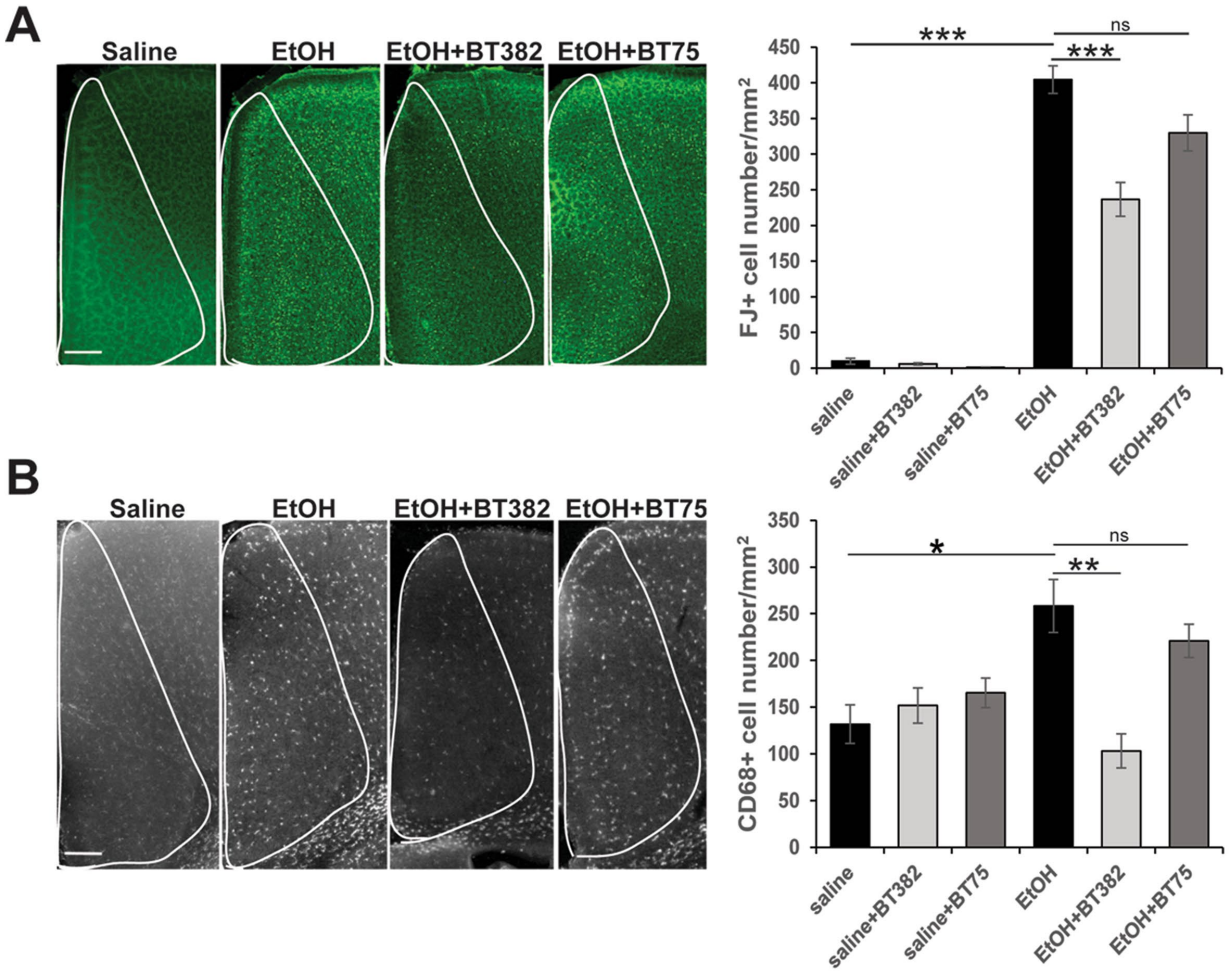
BT382 attenuated P7 ethanol-induced neurodegeneration. Mice were injected with saline/ethanol (EtOH) at P7, perfusion-fixed at P8 (20 h after injections), and the brain sections were stained with FJ **(A)** or immunofluorescence-stained using anti-CD68 antibody **(B)**. Images are representative brain sections in the cingulate cortex area (scale bar = 200 μm), and the graphs show FJ+ cell densities **(A)** and CD68+ cell densities **(B)** in the cingulate cortex (encompassed by white lines). *p < 0.05, **p < 0.01, ***p < 0.001 and ^ns^not significant by a Tukey test after one-way ANOVA. *n* = 4–5.

To examine the effects of BT382 (RARα antagonist) and BT75 (RARα agonist) on ethanol-induced acute neurodegeneration in neonatal mice, C57BL/6 J mice were injected (icv) with BT382 (25 μg/2 μl), BT75 (25 μg/2 μl), or the vehicle (10% DMSO/2 μl) 30 min before the first saline/ethanol injection (sc) at P7. Then, the mice were perfusion-fixed 20 h after the first saline/ethanol injection, when robust neurodegeneration has been observed (Olney et al., 2002b), and the brain sections were processed for detection of degenerating neurons by FJ staining. As reported previously (Sadrian et al., 2012), while FJ staining was very scarce in the saline controls, ethanol increased the density of FJ-positive (+) neurons in many brain regions including the cingulate cortex, retrosplenial cortex, and hippocampus. Figure 2A shows FJ+ cell densities in the cingulate cortex encompassed by white lines. Tukey HSD post-hoc test after one-way ANOVA indicated that the ethanol (EtOH) group is significantly different from the saline group, and also different from the EtOH+BT382 group, but not significantly different from EtOH+BT75 group, indicating significant reduction of neurodegeneration by BT382 but not by BT75.

Brain sections obtained for FJ staining were also fluorescence-labeled using anti-CD68 antibody, and CD68+ cell densities were counted in the cingulate cortex (Figure 2B). Similar to the results of FJ staining, Tukey HSD post-hoc test after one-way ANOVA indicated that the EtOH group was significantly different from the saline group and the EtOH+BT382 group, but not different from the EtOH+BT75 group, indicating significant reduction of CD68+ cells by BT382 but not by BT75. Our previous studies showed that after P7 ethanol treatment, Iba-1+ microglia found near dying neurons in the cortex became morphologically activated and subsequently phagocytosed FJ+ degenerated neurons, and these phagocytic microglia were co-localized with CD68+ cells (Saito et al., 2015). These studies suggest that CD68+ cells in the cingulate cortex in Figure 2B are activated phagocytic microglia, although some CD68+ phagocytic cells may be perivascular or invaded peripheral mononuclear macrophages. Thus, an RARα antagonist BT382 seems to attenuate P7 ethanol-induced acute neurodegeneration and the associated microglial activation and phagocytoses.

### P7 ethanol induced long-lasting GABA neuron reduction, which was partially attenuated by BT75

**Figure 3:**
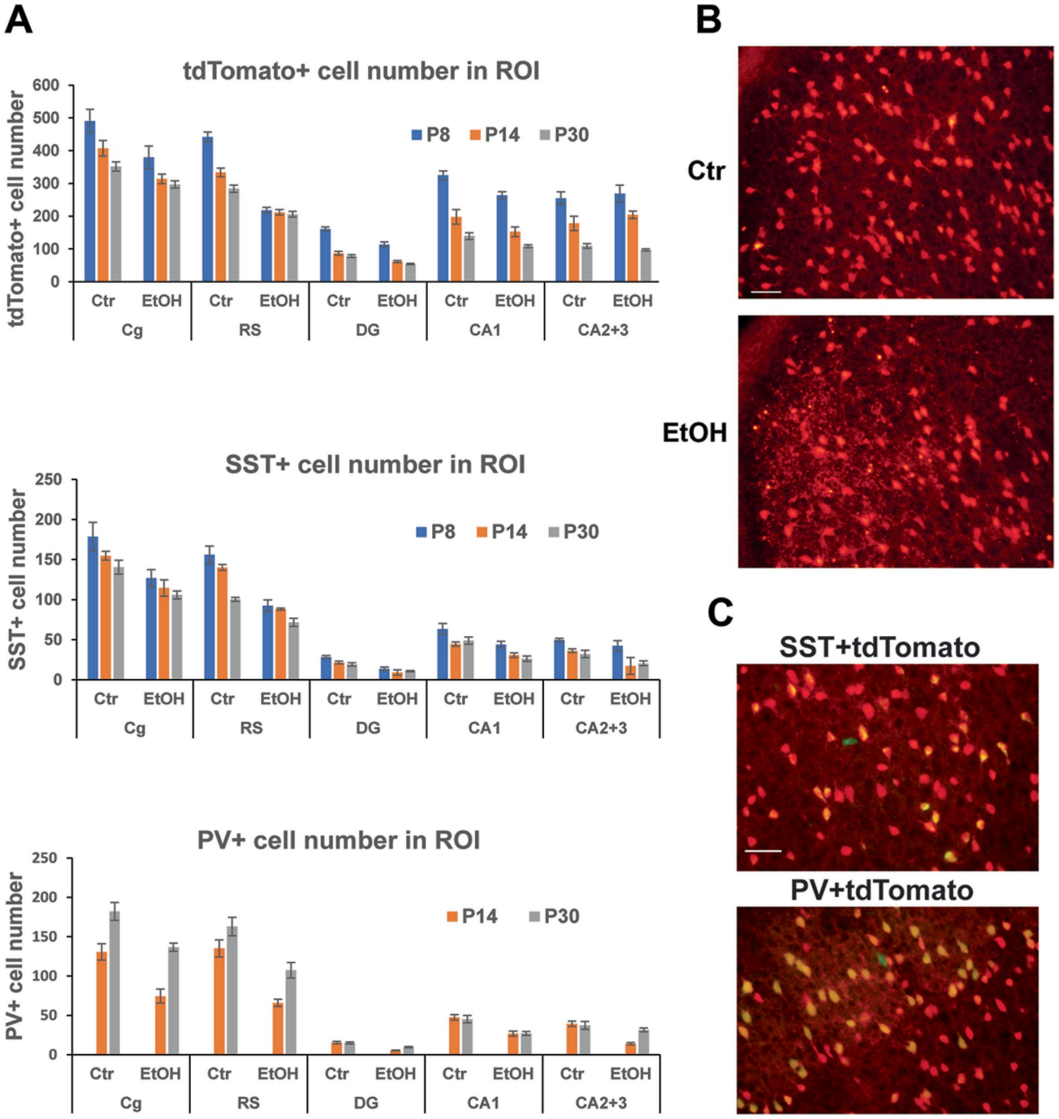
P7 ethanol induces long-lasting reduction in tdTomato+, SST+, and PV+ cells. **(A)** Nkx2.1-Cre;Ai9 mice were injected with saline (Ctr) or EtOH (2.5 g/kg, twice) at P7, and brains were perfusion-fixed at P8, P14, and P30 and vibratome-sectioned to count tdTomato+, SST+, and PV+ cell number in the cingulate cortex (Cg), retrosplenial cortex (RS), dorsal hippocampal dentate gyrus (DG), CA1, and CA2 + 3 regions by 2-dimensional counting. PV, which is barely expressed at P8 was only counted at P14 and P30. Mean ± SEM, *n* = 4. **(B)** 20 h after saline/EtOH treatment, Nkx2.1-Cre:Ai9 mice were perfusion-fixed, and brain sections were examined. EtOH treatment gave some cells neurodegenerative morphologies in RS. Scale bar = 50 μm. **(C)** PV+ cells (labeled green) and SST+ cells (green) were primarily co-localized with tdTomato+ cells (red) in the cortex at P30. Scale bar = 50 μm.

Our previous studies indicated that P7 ethanol, which induced acute neurodegeneration, also caused long-lasting reduction in PV+ and SST+ neurons in the cortex and hippocampus, and the reduction was more prominent than that of total neuron numbers (Smiley et al., 2015, 2019; Saito et al., 2019). However, since PV or SST cell numbers were estimated by counting PV+ or SST+ cells, it is not clear if the long-lasting reduction of those cells is due to P7 ethanol-induced acute neurodegeneration (cell loss) or reduction of PV/SST expression by disturbed cell maturation/homeostasis of surviving neurons. To ask this question, Nkx2.1-Cre;Ai9 mice were injected with ethanol (2.5 g/kg, twice) at P7, and brains taken at P8, P14, and P30 were vibratome-sectioned to examine tdTomato+ cells. The brain sections were also fluorescence-labeled using anti-SST or anti-PV antibody to detect PV+ or SST+ cells. Numbers of these labeled cells were estimated in the cingulate cortex (Cg), retrosplenial cortex (RS), dorsal hippocampal dentate gyrus (DG), CA1, and CA2 + CA3 (CA2 + 3) regions. Figure 3A shows cell numbers of tdTomato+, SST+, or PV+ cells of P8 (20 h after ethanol treatment), P14, and P30 mice after treatment with saline/EtOH at P7. Since PV expression was barely detectable at P8, PV+ cells were only counted at P14 and P30. Two-way ANOVA indicated that there were significant main effects of treatment (saline/EtOH) in the number of tdTomato+ neurons in Cg, RS, DG, and CA1, but not in CA2 + 3. Also, there were main effects of time points in all these ROIs, and there were significant interactions between the two factors (treatment and time points) in the RS and DG. In SST+ and PV+ cells, there were significant main effects of treatment in all regions measured including CA2 + 3 region. Also, there were significant main effects of time points in all regions in SST+ cells, and in Cg and RS regions in PV+ cells. The results indicate that P7 ethanol acutely eliminates significant amounts of tdTomato+ cells in the cortex, especially in RS and the degree of reduction is similar to that found in SST-labeled cells. Figure 3B shows a representative image of tdTomato+ cells in RS 20 h after saline (Ctr) or EtOH treatment, indicating that P7 ethanol induced degeneration in some of the tdTomato+ cells. This agrees with previous studies describing apoptotic cell death detected by caspase 3 activation in GABAergic interneurons in RS, 2–8 h after P7 ethanol treatment (Bird et al., 2020). Since most of the SST+ or PV+ cells in the RS at P30 are tdTomato+ cells as shown in Figure 3C, and the majority of tdTomato+ cells in the cortex are SST+, PV+, or their progenitors (Xu et al., 2008), it is likely that the loss of tdTomato+ cells at P8 is due to the loss of SST+, PV+, and their progenitors, considering the number of lost cells of tdTomato+ (~223) and SST+ cells (~64) in RS. The control (saline-treated) neurons also decreased during the period (P8-P30), and the differences in neuron numbers between the control and ethanol groups shrank. However, the apparent decrease in the cell number during the time course (P8-P30) may be due to our 2-dimensional cell counting method. To obtain total cell number in each brain region, stereological 3-dimensional counting is necessary.

Thus, acute neurodegeneration triggered by P7 ethanol may primarily induce long-time GABAergic cell loss especially in the cortex. However, considering the greater reduction of SST+ and PV+ neuron numbers than tdTomato+ cell numbers by P7 ethanol found in the hippocampus CA2 + 3, it is possible that P7 ethanol not only induces acute neurodegeneration, but also decreases SST and PV expression of surviving neurons and contributes to long-lasting GABAergic cell deficits.

**Figure 4:**
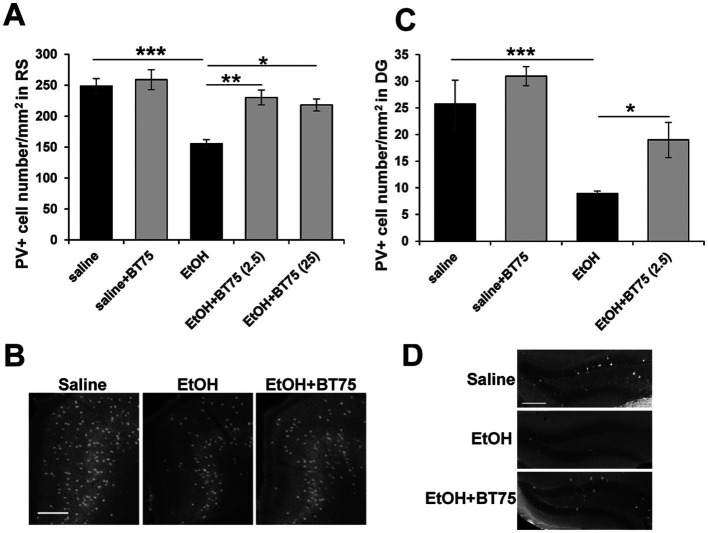
BT75 given before ethanol attenuates P7 ethanol-induced PV neuron deficits at P60. **(A)** 30 min before saline/EtOH administration, P7 mice were injected (icv) with 10% DMSO or BT75 (2.5 μg or 25 μg) and were perfusion-fixed at P60. Then, PV+ cell densities were counted in RS. ***p < 0.001, **p < 0.01, and *p < 0.05 by a Tukey post-hoc test after one-way ANOVA. *n* = 4. **(B)** Representative images used in **A**. **(C)** PV+ cell densities were counted in DG. ***p < 0.001 and *p < 0.05 by a Fisher’s LSD post-hoc test after one-way ANOVA. *n* = 4. **(D)** Representative images used in **C**. In **B** and **D**, scale bar = 200 μm.

Since RARα agonists and RA have been shown to have neuroprotective effects (Lee et al., 2009; Cai et al., 2019; Tian et al., 2022; Kang et al., 2023), and BT75 did not induce significant protection against acute neurodegeneration induced by P7 ethanol, we next examined if BT75 can affect long-lasting GABAergic cell deficits observed in the adult brain. C57BL/6 J mice were treated with BT75 and ethanol at P7, perfusion-fixed at P60, and the brain sections were used for PV immunohistochemistry. PV+ cell densities were counted in RS (Figures 4A,B) and DG (Figures 4C,D), where significant differences between saline and ethanol groups were detected in our previous studies (Lewin et al., 2018; Saito et al., 2019). In Figure 4A, Tukey HSD post-hoc test after one-way ANOVA indicated that the EtOH group was significantly different form the saline group and the EtOH+BT75 group. In Figure 4C, Fisher’s LSD post-hoc test after one-way ANOVA indicated that the EtOH group was significantly different form the saline group and the EtOH+BT75 group. These results indicate that BT75 partially restored PV+ neurons in these brain regions. Figure 4A also showed that 2.5 and 25 μg BT75 gave the similar neuroprotective effects.

### P7 ethanol induced long-lasting astrocyte activation, which was attenuated by BT75

**Figure 5:**
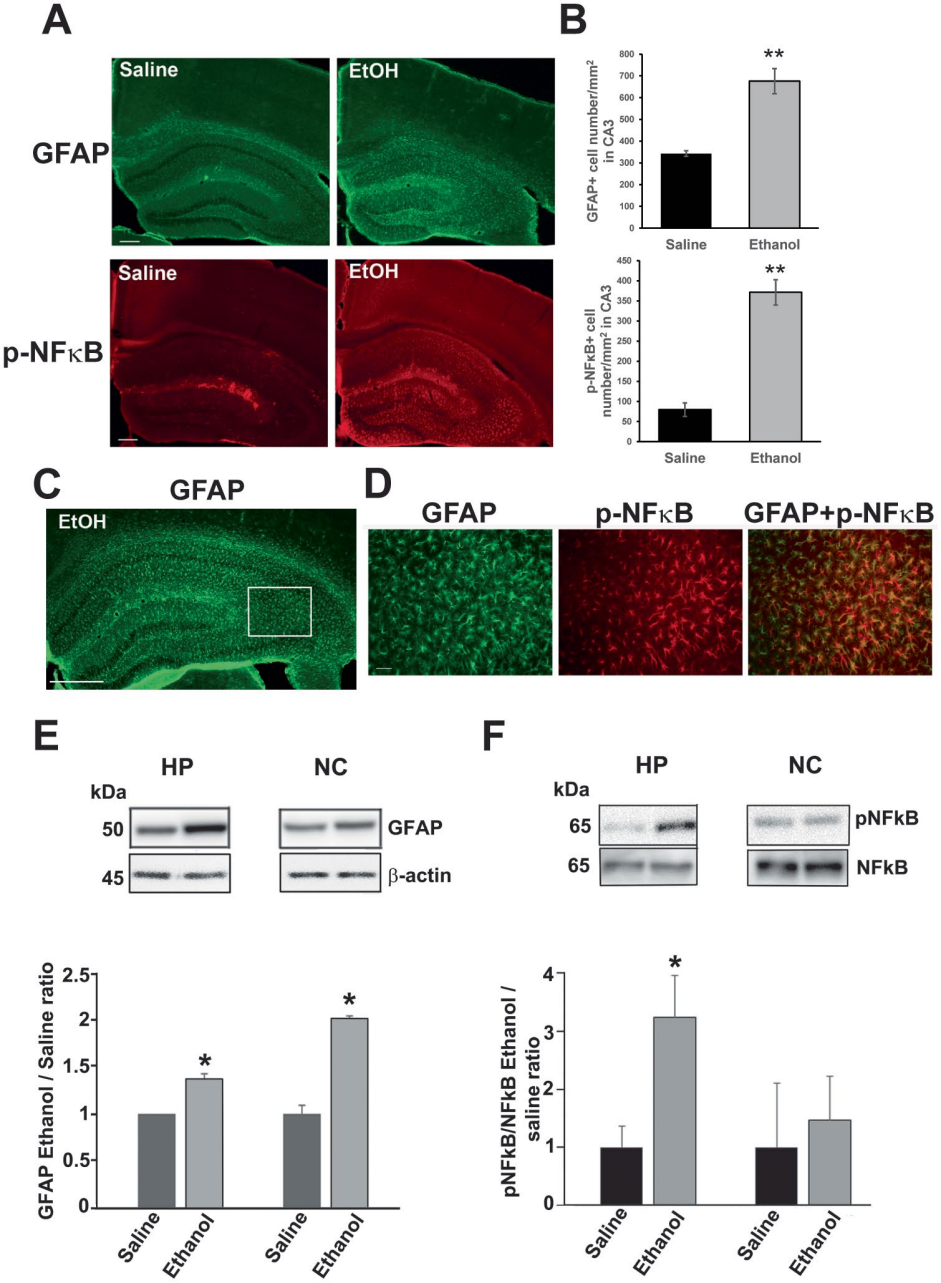
P7 ethanol increases GFAP and p-NFκB P65 levels at P90. **(A)** Mice injected with saline or ethanol at P7 were perfusion-fixed at P90, and the brain sections were immunostained by anti-GFAP antibody and anti-phospho-NFκB P65 (p-NFκB) antibody. Scale bar = 200 μm. **(B)** From the images, such as shown in **A**, GFAP+ and p-NFκB+ cell densities were measured in the dorsal hippocampal CA3 region. **p < 0.01 between saline and ethanol groups by Student’s *t*-test. *n* = 4. **(C,D)** Brain sections were dual-labeled with anti-GFAP antibody (green) and anti-p-NFκB antibody (red), and the images were overlaid. A rectangular region with white line (a part of the dorsal hippocampal CA3) in **C** was enlarged and shown in **D**. Scale bar = 500 μm in **C** and 50 μm in **D**. **(E)** Mice injected with saline or ethanol at P7 were sacrificed at P60, and the hippocampus (HP) and neocortex (NC) samples were used for Western blot analyzes of amounts of GFAP. The amounts of GFAP were normalized to actin and the ratio of ethanol/saline was calculated. *Significantly different by Student’s *t*-test. *n* = 4. **(F)** Similarly, the effects of P7 ethanol on pNFĸB expression were examined in hippocampus and cortex, The ratios of pNFĸB and NFĸB were compared as the ratios of ethanol/saline. *p < 0.05 by Student *t*-test. *n* = 4.

P7 ethanol also induces long-lasting astrocyte activation. As previously reported (Saito et al., 2015), P7 ethanol-induced neurodegeneration is followed by microglial activation which peaks around 24 h, and then astrocyte activation which peaks around 36 h. While microglial morphology returns to the resting states by 48 h after ethanol treatment, the densities of GFAP+ astrocytes are still significantly higher even in the adult brain. Figure 5A shows images of brain sections from P90 mice treated with saline or ethanol at P7. The brain sections were labeled using anti-GFAP (green) or anti-phospho-NFκB P65 (p-NFκB) (red) antibody. Using these images, labeled cell densities of dorsal hippocampus CA3 were measured (Figure 5B). Both GFAP+ cell densities and p-NFκB+ cell densities were significantly higher in ethanol-treated mice (Student’s *t*-test). Figure 5D indicates that p-NFκB+ cells were co-localized with GFAP+ cells. The area of Figure 5D is a part of dorsal hippocampal CA3 region, shown as a rectangle with white line in Figure 5C. Results of immunohistochemistry were confirmed by measuring the GFAP expression by Western blots using the hippocampus (HP) and neocortex (NC) of P60 mice treated with P7 saline/ethanol (Figure 5E). P7 ethanol also induced higher amounts of p-NFκB in the hippocampus but not in the cortex (Figure 5F).

**Figure 6:**
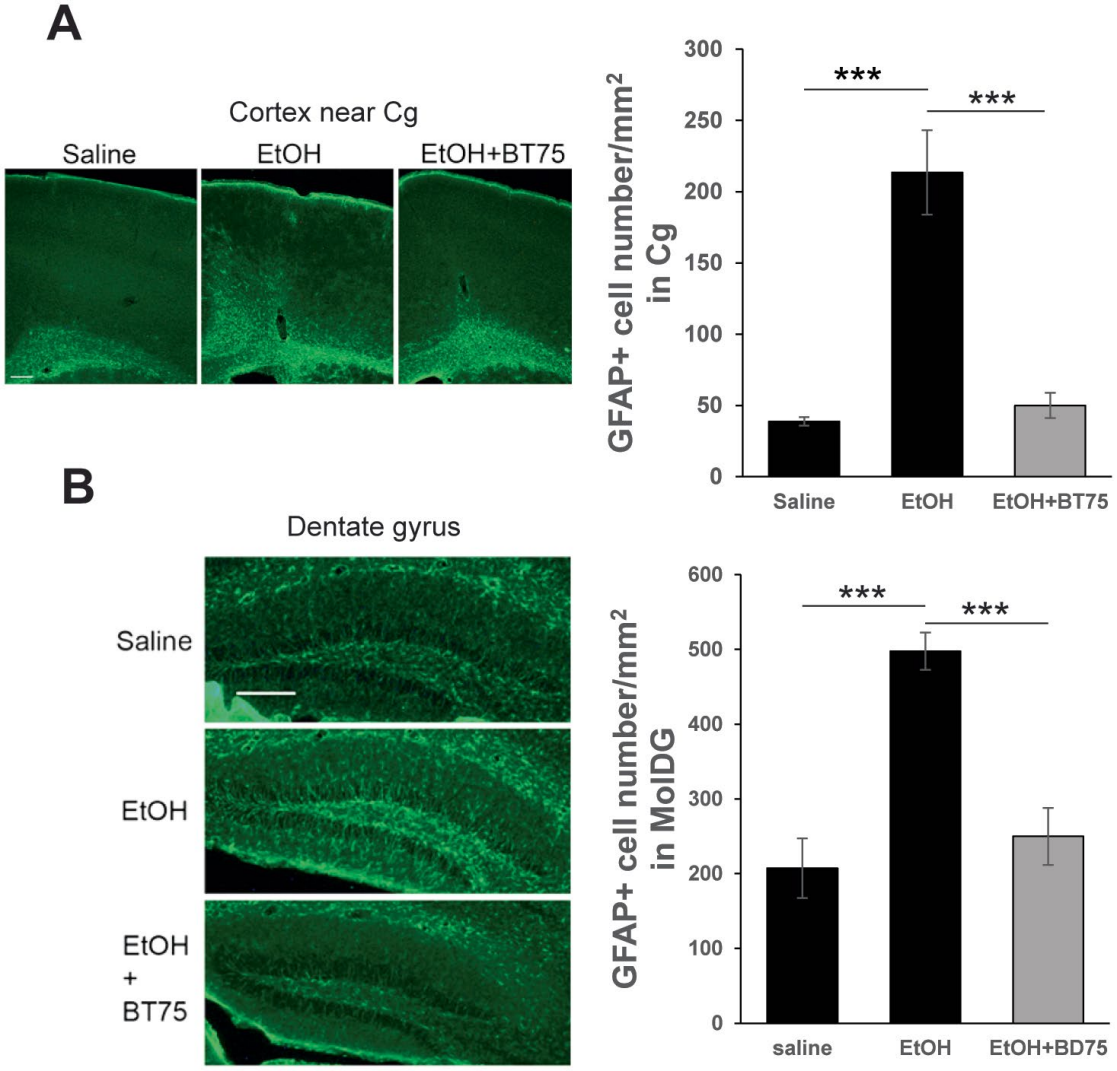
BT75 given before ethanol attenuates P7 ethanol-induced GFAP elevation at P60. **(A)** Mice were treated with BT75/vehicle and saline/EtOH at P7, and astrocyte activation was examined in the brain perfusion-fixed at P60. The brain sections were immunolabeled with anti-GFAP antibody. Scale bar = 200 μm. GFAP+ cell number was counted in the cingulate cortex (Cg). ***p < 0.001 by Tukey test after one-way ANOVA. *n* = 4. **(B)** GFAP+ cell number was counted in the molecular layers of dentate gyrus. ***p < 0.001 by Tukey test after one-way ANOVA. *n* = 4. Scale bar = 200 μm.

To examine the effects of BT75 on the long-lasting elevation GFAP+ cells by P7 ethanol, C57BL/6 J mice were treated with BT75 or the vehicle 30 min before saline/ethanol injections at P7, perfusion-fixed at P60, and the brain sections were examined by immunohistochemistry. As shown in Figure 6, densities of GFAP+ cells in the cingulate cortex (Cg) (A) or in the molecular layer of dentate gyrus (MolDG) (B) in the EtOH group were significantly higher than those in the saline group or those in the EtOH+BT75 group by Tukey HSD post-hoc test after one-way ANOVA.

### Post-ethanol administration of BT75 attenuated GABAergic cell deficits and astrocyte activation induced by P7 ethanol

**Figure 7:**
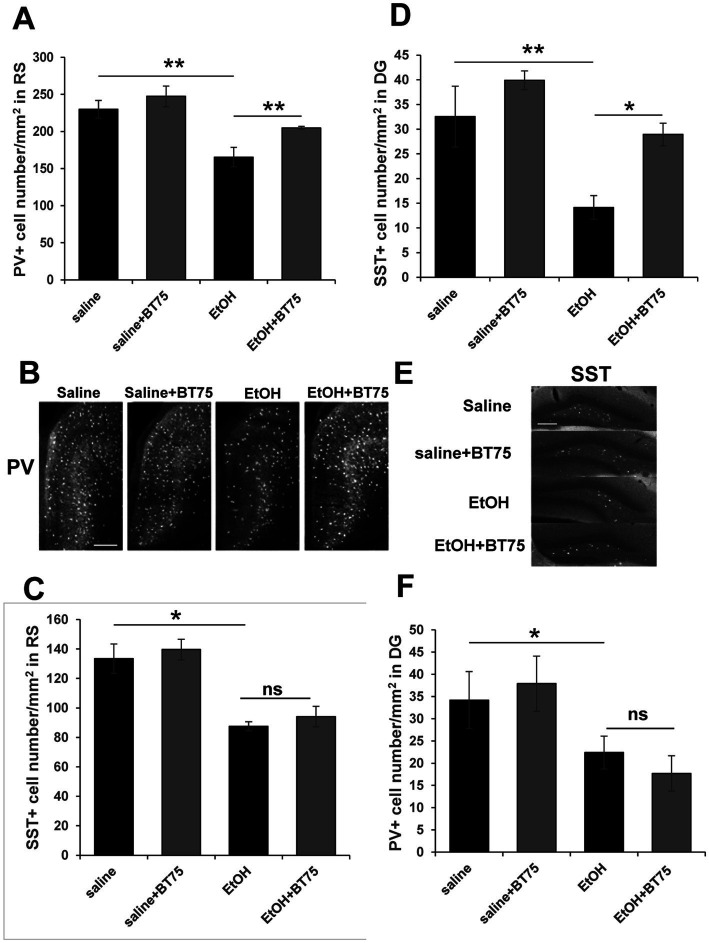
Post-ethanol treatment with BT75 attenuates P7 ethanol-induced GABA neuron deficits at P30. Three days after saline/ethanol injections at P7, mice were injected with BT75 (10 mg/kg) or the vehicle (10% DMSO) by ip once a day for 3 days. Then at P30, mice were perfusion-fixed and the brain sections were immunolabeled using anti-PV or anti-SST antibody. **(A)** PV+ cell density in RS. **p < 0.01 and *p < 0.05 by a Fisher’s LSD post-hoc test after one-way ANOVA. *n* = 4. **(B)** Representative images used for **A**. **(C)** SST+ cell densities in RS. *p < 0.05 and ^ns^not significant by a Fisher’s LSD post-hoc test after one-way ANOVA. *n* = 4. **(D)** SST+ cell densities in DG. **p < 0.01 and *p < 0.05 by a Tukey post-hoc test after one-way ANOVA. *n* = 4. **(E)** Representative images used for **D**. **(F)** PV+ cell densities in DG. *p < 0.05 and ^ns^not significant by a Fisher’s LSD post-hoc test after one-way ANOVA. *n* = 4. In **B** and **E**, scale bar = 200 μm.

**Figure 8:**
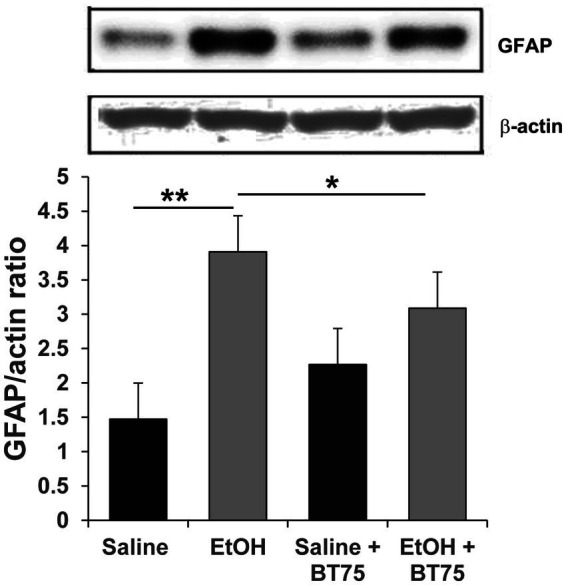
Post-ethanol treatment with BT75 attenuates P7 ethanol-induced GFAP elevation at P30. Mice were treated with BT75/vehicle (10 mg/kg, once a day for 3 days) starting 3 days after saline/EtOH injections at P7, and the hippocampus was dissected at P30 and the ratio of GFAP/actin was measured by Western blot analyzes. BT75 significantly reduced astrocyte activation induced by EtOH. *p < 0.05, **p < 0.001, *n* = 4.

Next, we examined if BT75 can attenuate P7 ethanol-induced GABAergic neuronal deficits or astrocyte activation, when BT75 is applied after ethanol administration. Three days after saline/ethanol injections at P7, BT75 were injected (ip) once a day for 3 days. At P30, the mice were perfusion-fixed, and PV+ or SST+ cell densities were counted in RS and DG. Figures 7A,B show that PV+ cell density in RS of EtOH group is significantly different from the saline group and EtOH+BT75 group by a Fisher’s LSD post-hoc test after one-way ANOVA. However, as shown in Figure 7C, SST+ cell densities in RS of EtOH group, which is significantly different from saline group, is not different from EtOH+BT75 group by a LSD post-hoc test after one-way ANOVA. On the contrary, BT75 significantly (by Tukey HSD post-hoc test after one-way ANOVA) attenuated SST+ cell reduction in DG (Figures 7D,E), but did not attenuate PV+ cell reduction in DG by a Fisher’s LSD post-hoc test) (Figure 7F). We also assessed if post-ethanol treatment by BT75 reduces elevation of GFAP expression. After mice were treated as described above, the hippocampus was dissected out at P30, and GFAP levels were analyzed by Western blots (Figure 8). The result shows that post-ethanol treatment by BT75 partially attenuated GFAP expression in the hippocampus, which was enhanced by P7 ethanol.

Thus, BT75 injected after ethanol treatment still attenuated P7 ethanol-induced GABAergic cell deficits and astrocyte activation in certain brain regions, which may emphasize the possibility that some of the GABAergic cell deficits induced by P7 ethanol are reversible.

## Discussion

Previous studies have shown that ethanol induces robust neurodegeneration within 24 h in many brain regions of P7 mice (Olney et al., 2002a; Saito et al., 2007), and the reduction in number of GABAergic cells (mainly PV+ and SST+ neurons) in the cortex and hippocampus remains evident in the adult brain (Smiley et al., 2015, 2019; Saito et al., 2019), which may partially explain behavioral deficits observed in P7 ethanol-treated mice (Sadrian et al., 2012, 2014; Wilson et al., 2016; Lewin et al., 2018). Present studies indicated that an RARα specific antagonist BT382 and an agonist BT75 attenuated ethanol-induced cellular abnormalities in the developing brain in different manners. BT382 attenuated P7 ethanol-induced acute neurodegeneration detected by FJ staining 20 h after ethanol injection. Along with it, BT382 decreased the density of CD68+ cells. Our previous studies (Saito et al., 2015) have indicated that these CD68+ cells are co-localized with activated Iba-1+ cells which surround and phagocytose FJ+ degenerating neurons. These CD68 + Iba-1+ cells seem to be phagocytic microglia although some of these cells can be perivascular or invaded peripheral mononuclear macrophages. These results indicate that BT382 attenuated P7 ethanol-induced apoptotic neurodegeneration and the subsequent microglial activation, although we may need a time course study because BT382 may only shift the timing of neurodegeneration. Presently, mechanisms of neuroprotection by BT382 are unknown, but the toxic effects of excess RA may be reduced by the RARα antagonist because it has been reported that prenatal ethanol treatment increased ATRA in fetal hippocampus and cortex (Kane et al., 2010). Previous studies have also indicated that chaperon-mediated autophagy (CMA), which is inhibited by RARα, is activated by BT382 without inhibiting macroautophagy (Anguiano et al., 2013; Gomez-Sintes et al., 2022). CMA activation, which shows neuroprotection in neurodegenerative diseases and acute neurological insults (Cuervo and Wong, 2014; Kanno et al., 2022), may be related to the neuroprotective effects of BT382 against P7 ethanol toxicity. Presently, we have not studied the long-term effects of BT382, partly because we expect that once initial apoptotic neurodegeneration is blocked, subsequent glial activation and GABAergic cell loss would be also blocked, as indicated in our previous studies using lithium (Sadrian et al., 2012; Lewin et al., 2018; Saito et al., 2019). However, studies on long-term effects of BT382, especially post-ethanol treatment by BT382 may be necessary to evaluate the efficacy of BT382 as a neuroprotective agent and to elucidate mechanisms of its action.

In contrast to the effects of BT382, RARα agonist BT75 did not affect the acute neurodegeneration but reduced long lasting GABAergic cell loss and chronic astrocyte activation induced by P7 ethanol. The long-term deficits of GABAergic neurons may be caused by acute apoptotic neurodegeneration at P7 and/or by delayed cell loss or disturbances in surviving neurons, which may lower expression of PV or SST. Studies using Nkx2.1-Cre;Ai9 mice (Figure 3A) showed that tdTomato+ cell numbers were reduced by ethanol in a similar manner as SST+ cells in the Cg and RS, indicating SST cell deficits observed in the adult cortex is mainly due to acute apoptotic neurodegeneration. The cell death that occurred in PV+ neurons at P7-P8 was not directly measured because of the lack of PV expression at this time. However, the reduction in the number of tdTomato+ cells by ethanol in Cg or RS at P8 indicates that not only SST+ cells but also PV cell progenitors were decreased by acute neurodegeneration. However, in the hippocampus, effects of P7 ethanol are different between SST+ cells and tdTomato+ cells. Especially in CA2 and 3 regions, while tdTomato+ cell numbers were not significantly reduced by P7 ethanol at P30, SST+ or PV+ cell numbers were significantly reduced by ethanol. These results indicate that long-term reduction in SST+ or PV+ cells is due to acute neurodegeneration as well as impaired maturation or homeostasis of surviving neurons as suggested in our previous studies (Saito et al., 2019). Our results showing that BT75 increased PV+ or SST+ neurons in certain brain regions even though BT75 was given after P7 ethanol-induced neurodegeneration (Figure 7), also suggest that BT75 may restore the expression of PV or SST in some surviving neurons. Our previous experiments indicated that other neuroprotective treatments [lithium (Saito et al., 2019) and environmental enrichment/exercise (Apuzzo et al., 2020)] given after P7 ethanol treatment also partially restored the long-term PV+ neuron deficits induced by P7 ethanol. Interestingly, the decrease in PV+ neurons in the dentate gyrus by P7 ethanol was not rescued in all these post-ethanol treatments. Thus, these post-ethanol neuroprotective effects seem to be cell or brain region-specific. Mechanisms of such restoration of PV or SST neurons by BT75 is not clear. It may be related to previous studies indicating that RA signaling plays an important role in early postnatal development of prefrontal PV neurons (Larsen et al., 2019) and that RA regulates PV expression in the mouse model of ischemic stroke (Kang et al., 2023). Alternatively, the neuroprotective effects of BT75 may be related to its anti-inflammatory action. P7 ethanol induces chronic astrocyte activation detected by increases in GFAP+ cells in immunohistochemistry (Figure 6) or by increases in GFAP expression in Western blot (Figure 8). Such GFAP elevation was reduced by either pre–(Figure 6) or post-ethanol (Figure 8) BT75 administration. It has been also reported that RARα agonists show anti-inflammatory activity in both cultured cells and animal models (Tian et al., 2022). ATRA inhibits expression of TNFα and iNOS in primary rat microglia activated by LPS or Aβ (Dheen et al., 2005) and attenuates neuroinflammation induced by ethanol in adult rat brain by modulating SIRT1 and NF-κB (Priyanka et al., 2018). Our previous studies also indicate that BT75 is a potent anti-inflammatory agent in SIM-A9 microglial cell line activated with LPS (Zhang et al., 2023). Since excessive or chronic neuroinflammation has been implicated in ethanol-induced neurodegeneration (Chastain and Sarkar, 2014; Pascual et al., 2017; Zhang et al., 2018; Saito et al., 2019; Kane and Drew, 2021), anti-inflammatory action of BD75 may exert neuroprotection of PV and SST neurons.

Thus, our studies suggest the involvement of RA signaling in neonatal ethanol-induced neurodegeneration and neuroinflammation. RA/RARα signaling may serve as a target to improve developmental ethanol-induced brain abnormalities observed in FASD patients.

## Data availability statement

The original contributions presented in the study are included in the article/supplementary material, further inquiries can be directed to the corresponding authors.

## Ethics statement

The animal study was reviewed and approved by the Nathan Kline Institute Institutional Animal Care and Use Committee.

## Author contributions

MS involved in the study design and conception, data acquisition and analysis, and writing the manuscript. SS, XZ, and SC-B engaged in data acquisition and analyzes and commenting and editing the drafts. JS and DW engaged in the study design and conception and commenting and editing the drafts. BD engaged in syntheses of BT75 and BT382, involved in the study design and conception and commenting and editing the drafts. All authors contributed to the article and approved the submitted version.

## Funding

This work was supported by grants from National Institute on Alcohol Abuse and Alcoholism (R21AA027374 to BD and MS; R01AA023181 to MS, DW, and JS).

## Conflict of interest

The authors declare that the research was conducted in the absence of any commercial or financial relationships that could be construed as a potential conflict of interest.

## Publisher’s note

All claims expressed in this article are solely those of the authors and do not necessarily represent those of their affiliated organizations, or those of the publisher, the editors and the reviewers. Any product that may be evaluated in this article, or claim that may be made by its manufacturer, is not guaranteed or endorsed by the publisher.

## References

[ref1] Alfos S. Boucheron C. Pallet V. Higueret D. Enderlin V. Béracochéa D.. (2001). A retinoic acid receptor antagonist suppresses brain retinoic acid receptor overexpression and reverses a working memory deficit induced by chronic ethanol consumption in mice. Alcohol. Clin. Exp. Res. 25, 1506–1514. doi: 10.1111/j.1530-0277.2001.tb02154.x, PMID: 11696672

[ref2] Anguiano J. Garner T. P. Mahalingam M. Das B. C. Gavathiotis E. Cuervo A. M. (2013). Chemical modulation of chaperone-mediated autophagy by retinoic acid derivatives. Nat. Chem. Biol. 9, 374–382. doi: 10.1038/nchembio.1230, PMID: 23584676 PMC3661710

[ref3] Apuzzo J. Saito M. Wilson D. A. (2020). Post-exposure environment modulates long-term developmental ethanol effects on behavior, neuroanatomy, and cortical oscillations. Brain Res. 1748:147128. doi: 10.1016/j.brainres.2020.147128, PMID: 32950485 PMC7572797

[ref4] Astley S. J. Aylward E. H. Olson H. C. Kerns K. Brooks A. Coggins T. E.. (2009). Magnetic resonance imaging outcomes from a comprehensive magnetic resonance study of children with fetal alcohol spectrum disorders. Alcohol. Clin. Exp. Res. 33, 1671–1689. doi: 10.1111/j.1530-0277.2009.01004.x, PMID: 19572986 PMC4170878

[ref5] Bird C. W. Barber M. J. Post H. R. Jacquez B. Chavez G. J. Faturos N. G.. (2020). Neonatal ethanol exposure triggers apoptosis in the murine retrosplenial cortex: role of inhibition of NMDA receptor-driven action potential firing. Neuropharmacology 162:107837. doi: 10.1016/j.neuropharm.2019.107837, PMID: 31689422 PMC6899211

[ref6] Bird C. W. Taylor D. H. Pinkowski N. J. Chavez G. J. Valenzuela C. F. (2018). Long-term reductions in the population of GABAergic interneurons in the mouse hippocampus following developmental ethanol exposure. Neuroscience 383, 60–73. doi: 10.1016/j.neuroscience.2018.05.003, PMID: 29753864 PMC5994377

[ref7] Cai W. Wang J. Hu M. Chen X. Lu Z. Bellanti J. A.. (2019). All trans-retinoic acid protects against acute ischemic stroke by modulating neutrophil functions through STAT1 signaling. J. Neuro-Oncol. 16:175. doi: 10.1186/s12974-019-1557-6, PMID: 31472680 PMC6717357

[ref8] Chastain L. G. Sarkar D. K. (2014). Role of microglia in regulation of ethanol neurotoxic action. Int. Rev. Neurobiol. 118, 81–103. doi: 10.1016/B978-0-12-801284-0.00004-X, PMID: 25175862

[ref9] Coleman L. G. Oguz I. Lee J. Styner M. Crews F. T. (2012). Postnatal day 7 ethanol treatment causes persistent reductions in adult mouse brain volume and cortical neurons with sex specific effects on neurogenesis. Alcohol 46, 603–612. doi: 10.1016/j.alcohol.2012.01.003, PMID: 22572057 PMC3552379

[ref10] Crandall J. E. Goodman T. McCarthy D. M. Duester G. Bhide P. G. Dräger U. C.. (2011). Retinoic acid influences neuronal migration from the ganglionic eminence to the cerebral cortex. J. Neurochem. 119, 723–735. doi: 10.1111/j.1471-4159.2011.07471.x, PMID: 21895658 PMC3732058

[ref11] Cuervo A. M. Wong E. (2014). Chaperone-mediated autophagy: roles in disease and aging. Cell Res. 24, 92–104. doi: 10.1038/cr.2013.153, PMID: 24281265 PMC3879702

[ref12] Das B. C. Mohapatra S. Campbell P. D. Nayak S. Mahalingam S. M. Evans T. (2010). Synthesis of function-oriented 2-phenyl-2H-chromene derivatives using L-pipecolinic acid and substituted guanidine organocatalysts. Tetrahedron Lett. 51, 2567–2570. doi: 10.1016/j.tetlet.2010.02.143, PMID: 21785516 PMC3140427

[ref13] Dheen S. T. Jun Y. Yan Z. Tay S. S. Ling E. A. (2005). Retinoic acid inhibits expression of TNF-alpha and iNOS in activated rat microglia. Glia 50, 21–31. doi: 10.1002/glia.20153, PMID: 15602748

[ref14] Dowling J. E. (2020). Vitamin a: its many roles-from vision and synaptic plasticity to infant mortality. J. Comp. Physiol. A Neuroethol. Sens Neural Behav. Physiol. 206, 389–399. doi: 10.1007/s00359-020-01403-z, PMID: 32034476

[ref15] Duester G. (1991). A hypothetical mechanism for fetal alcohol syndrome involving ethanol inhibition of retinoic acid synthesis at the alcohol dehydrogenase step. Alcohol. Clin. Exp. Res. 15, 568–572. doi: 10.1111/j.1530-0277.1991.tb00562.x, PMID: 1877746

[ref16] Fainsod A. Bendelac-Kapon L. Shabtai Y. (2020). Alcohol Spectrum disorder: embryogenesis under reduced retinoic acid signaling conditions. Subcell. Biochem. 95, 197–225. doi: 10.1007/978-3-030-42282-0_8, PMID: 32297301

[ref17] Gomez-Sintes R. Xin Q. Jimenez-Loygorri J. I. McCabe M. Diaz A. Garner T. P.. (2022). Targeting retinoic acid receptor alpha-corepressor interaction activates chaperone-mediated autophagy and protects against retinal degeneration. Nat. Commun. 13:4220. doi: 10.1038/s41467-022-31869-1, PMID: 35864098 PMC9304322

[ref18] Haushalter C. Asselin L. Fraulob V. Dollé P. Rhinn M. (2017). Retinoic acid controls early neurogenesis in the developing mouse cerebral cortex. Dev. Biol. 430, 129–141. doi: 10.1016/j.ydbio.2017.08.006, PMID: 28790015

[ref19] Johnson C. S. Zucker R. M. Hunter E. S. 3rd Sulik K. K. (2007). Perturbation of retinoic acid (RA)-mediated limb development suggests a role for diminished RA signaling in the teratogenesis of ethanol. Birth Defects Res. A Clin. Mol. Teratol. 79, 631–641. doi: 10.1002/bdra.20385, PMID: 17676605

[ref20] Kane C. J. M. Drew P. D. (2021). Neuroinflammatory contribution of microglia and astrocytes in fetal alcohol spectrum disorders. J. Neurosci. Res. 8, 1973–1985. doi: 10.1002/jnr.24735 PMC8147698 32959429

[ref21] Kane M. A. Folias A. E. Wang C. Napoli J. L. (2010). Ethanol elevates physiological all-trans-retinoic acid levels in select loci through altering retinoid metabolism in multiple loci: a potential mechanism of ethanol toxicity. FASEB J. 24, 823–832. doi: 10.1096/fj.09-141572, PMID: 19890016 PMC2830136

[ref22] Kang J. B. Park D. J. Koh P. O. (2023). Retinoic acid prevents the neuronal damage through the regulation of Parvalbumin in an ischemic stroke model. Neurochem. Res. 48, 487–501. doi: 10.1007/s11064-022-03769-9, PMID: 36245066

[ref23] Kanno H. Handa K. Murakami T. Aizawa T. Ozawa H. (2022). Chaperone-mediated autophagy in neurodegenerative diseases and acute neurological insults in the central nervous system. Cells 11:1205. doi: 10.3390/cells11071205, PMID: 35406769 PMC8997510

[ref24] Larsen R. Proue A. Scott E. P. Christiansen M. Nakagawa Y. (2019). The thalamus regulates retinoic acid signaling and development of Parvalbumin interneurons in postnatal mouse prefrontal cortex. eNeuro 6:18. doi: 10.1523/ENEURO.0018-19.2019 PMC6385081 30868103

[ref25] Lee H. P. Casadesus G. Zhu X. Lee H. G. Perry G. Smith M. A.. (2009). All-trans retinoic acid as a novel therapeutic strategy for Alzheimer’s disease. Expert. Rev. Neurother. 9, 1615–1621. doi: 10.1586/ern.09.86, PMID: 19903021 PMC2913310

[ref26] Lewin M. Ilina M. Betz J. Masiello K. Hui M. Wilson D. A.. (2018). Developmental ethanol-induced sleep fragmentation, behavioral hyperactivity, cognitive impairment and Parvalbumin cell loss are prevented by lithium co-treatment. Neuroscience 369, 269–277. doi: 10.1016/j.neuroscience.2017.11.033, PMID: 29183826 PMC5766420

[ref27] Marrs J. A. Clendenon S. G. Ratcliffe D. R. Fielding S. M. Liu Q. Bosron W. F. (2010). Zebrafish fetal alcohol syndrome model: effects of ethanol are rescued by retinoic acid supplement. Alcohol 44, 707–715. doi: 10.1016/j.alcohol.2009.03.004, PMID: 20036484 PMC2889201

[ref28] Mattioli I. Sebald A. Bucher C. Charles R. P. Nakano H. Doi T.. (2004). Transient and selective NF-kappa B p65 serine 536 phosphorylation induced by T cell costimulation is mediated by I kappa B kinase beta and controls the kinetics of p65 nuclear import. J. Immunol. 172:6336-44. doi: 10.4049/jimmunol.172.10.6336, PMID: 15128824

[ref29] May P. A. Baete A. Russo J. Elliott A. J. Blankenship J. Kalberg W. O.. (2014). Prevalence and characteristics of fetal alcohol spectrum disorders. Pediatrics 134, 855–866. doi: 10.1542/peds.2013-3319, PMID: 25349310 PMC4210790

[ref30] May P. A. Gossage J. P. Kalberg W. O. Robinson L. K. Buckley D. Manning M.. (2009). Prevalence and epidemiologic characteristics of FASD from various research methods with an emphasis on recent in-school studies. Dev. Disabil. Res. Rev. 15, 176–192. doi: 10.1002/ddrr.68, PMID: 19731384

[ref31] McCaffery P. Koul O. Smith D. Napoli J. L. Chen N. Ullman M. D. (2004). Ethanol increases retinoic acid production in cerebellar astrocytes and in cerebellum. Brain Res. Dev. Brain Res. 153, 233–241. doi: 10.1016/j.devbrainres.2004.09.003, PMID: 15527891

[ref32] Mey J. Mccaffery P. (2004). Retinoic acid signaling in the nervous system of adult vertebrates. Neuroscientist 10, 409–421. doi: 10.1177/1073858404263520 15359008

[ref33] Niederreither K. Dollé P. (2008). Retinoic acid in development: towards an integrated view. Nat. Rev. Genet. 9, 541–553. doi: 10.1038/nrg2340, PMID: 18542081

[ref34] Norman A. L. Crocker N. Mattson S. N. Riley E. P. (2009). Neuroimaging and fetal alcohol spectrum disorders. Dev. Disabil. Res. Rev. 15, 209–217. doi: 10.1002/ddrr.72, PMID: 19731391 PMC3442778

[ref35] Olney J. W. Tenkova T. Dikranian K. Muglia L. J. Jermakowicz W. J. D’Sa C.. (2002b). Ethanol-induced caspase-3 activation in the in vivo developing mouse brain. Neurobiol. Dis. 9, 205–219. doi: 10.1006/nbdi.2001.0475, PMID: 11895372

[ref36] Olney J. W. Tenkova T. Dikranian K. Qin Y. Q. Labruyere J. Ikonomidou C. (2002a). Ethanol-induced apoptotic neurodegeneration in the developing C57BL/6 mouse brain. Brain Res. Dev. Brain Res. 133, 115–126. doi: 10.1016/S0165-3806(02)00279-1, PMID: 11882342

[ref37] Pascual M. Montesinos J. Montagud-Romero S. Forteza J. Rodríguez-Arias M. Miñarro J.. (2017). TLR4 response mediates ethanol-induced neurodevelopment alterations in a model of fetal alcohol spectrum disorders. J. Neuroinflammation 14:145. doi: 10.1186/s12974-017-0918-2, PMID: 28738878 PMC5525270

[ref38] Paxinos G. Franklin K. B. J.. (2004). The mouse brain in stereotaxic coordinates. New York: Elsevier, Academic Press.

[ref39] Paxinos G. Halliday G. Watson C. Koutcherov Y. Wang H.. (2007) Atlas of the developing mouse brain at E17.5, P0, and P6. New York: Elsevier, Academic Press.

[ref40] Priyanka S. H. Syam Das S. Thushara A. J. Rauf A. A. Indira M. (2018). All trans retinoic acid attenuates markers of Neuroinflammation in rat brain by modulation of SIRT1 and NFκB. Neurochem. Res. 43, 1791–1801. doi: 10.1007/s11064-018-2595-7, PMID: 30022380

[ref41] Rhinn M. Dollé P. (2012). Retinoic acid signalling during development. Development 139, 843–858. doi: 10.1242/dev.065938, PMID: 22318625

[ref42] Riley E. P. Infante M. A. Warren K. R. (2011). Fetal alcohol spectrum disorders: an overview. Neuropsychol. Rev. 21, 73–80. doi: 10.1007/s11065-011-9166-x, PMID: 21499711 PMC3779274

[ref43] Riley E. P. McGee C. L. (2005). Fetal alcohol spectrum disorders: an overview with emphasis on changes in brain and behavior. Exp. Biol. Med. (Maywood) 230, 357–365. doi: 10.1177/15353702-0323006-03, PMID: 15956765

[ref44] Sadakata T. Washida M. Iwayama Y. Shoji S. Sato Y. Ohkura T.. (2007). Autistic-like phenotypes in Cadps2-knockout mice and aberrant CADPS2 splicing in autistic patients. J. Clin. Invest. 117, 931–943. doi: 10.1172/JCI29031, PMID: 17380209 PMC1821065

[ref45] Sadrian B. Lopez-Guzman M. L. Wilson D. A. Saito M. (2014). Distinct neurobehavioral dysfunction based on the timing of developmental binge-like alcohol exposure. Neuroscience 280, 204–219. doi: 10.1016/j.neuroscience.2014.09.008, PMID: 25241068 PMC4250396

[ref46] Sadrian B. Subbanna S. Wilson D. A. Basavarajappa B. S. Saito M. (2012). Lithium prevents long-term neural and behavioral pathology induced by early alcohol exposure. Neuroscience 206, 122–135. doi: 10.1016/j.neuroscience.2011.12.059, PMID: 22266347 PMC3294020

[ref47] Saito M. Mao R. F. Wang R. Vadasz C. Saito M. (2007). Effects of gangliosides on ethanol-induced neurodegeneration in the developing mouse brain. Alcohol. Clin. Exp. Res. 31, 665–674. doi: 10.1111/j.1530-0277.2007.00351.x, PMID: 17374046

[ref48] Saito M. Saito M. Das B. C. (2019). Involvement of AMP-activated protein kinase in neuroinflammation and neurodegeneration in the adult and developing brain. Int. J. Dev. Neurosci. 77, 48–59. doi: 10.1016/j.ijdevneu.2019.01.007, PMID: 30707928 PMC6663660

[ref49] Saito M. Smiley J. F. Hui M. Masiello K. Betz J. Ilina M.. (2019). Neonatal ethanol disturbs the Normal maturation of Parvalbumin interneurons surrounded by subsets of Perineuronal nets in the cerebral cortex: partial reversal by lithium. Cereb. Cortex 29, 1383–1397. doi: 10.1093/cercor/bhy034, PMID: 29462278 PMC6418394

[ref50] Saito M. Wu G. Hui M. Masiello K. Dobrenis K. Ledeen R. W.. (2015). Ganglioside accumulation in activated glia in the developing brain: comparison between WT and GalNAc KO mice. J. Lipid Res. 56, 1434–1448. doi: 10.1194/jlr.M056580, PMID: 26063460 PMC4513985

[ref51] Sapin V. (2008). Alcohol and pregnancy: diagnostic aspects and abnormalities in the placental vitamin a pathway. Ann. Biol. Clin. (Paris) 66, 509–513. doi: 10.1684/abc.2008.0271, PMID: 18957339

[ref52] Shabtai Y. Bendelac L. Jubran H. Hirschberg J. Fainsod A. (2018). Acetaldehyde inhibits retinoic acid biosynthesis to mediate alcohol teratogenicity. Sci. Rep. 8:347. doi: 10.1038/s41598-017-18719-7, PMID: 29321611 PMC5762763

[ref53] Shearer K. Stoney P. N. Morgan P. J. McCaffery P. J. (2012). A vitamin for the brain. Trends Neurosci. 35, 733–741. doi: 10.1016/j.tins.2012.08.005 22959670

[ref54] Smiley J. F. Bleiwas C. Masiello K. Petkova E. Betz J. Hui M.. (2019). Effects of neonatal ethanol on cerebral cortex development through adolescence. Brain Struct. Funct. 224, 1871–1884. doi: 10.1007/s00429-019-01881-1, PMID: 31049690 PMC6565455

[ref55] Smiley J. F. Saito M. Bleiwas C. Masiello K. Ardekani B. Guilfoyle D. N.. (2015). Selective reduction of cerebral cortex GABA neurons in a late gestation model of fetal alcohol spectrum disorder. Alcohol 49, 571–580. doi: 10.1016/j.alcohol.2015.04.008, PMID: 26252988 PMC4554880

[ref56] Tian Y. Liu B. Li Y. Zhang Y. Shao J. Wu P.. (2022). Activation of RARα receptor attenuates Neuroinflammation after SAH via promoting M1-to-M2 phenotypic polarization of microglia and regulating Mafb/Msr1/PI3K-Akt/NF-κB pathway. Front. Immunol. 13:839796. doi: 10.3389/fimmu.2022.839796, PMID: 35237277 PMC8882645

[ref57] Wilson D. A. Masiello K. Lewin M. P. Hui M. Smiley J. F. Saito M. (2016). Developmental ethanol exposure-induced sleep fragmentation predicts adult cognitive impairment. Neuroscience 322, 18–27. doi: 10.1016/j.neuroscience.2016.02.020, PMID: 26892295 PMC4805438

[ref58] Wolf G. (2010). Tissue-specific increases in endogenous all-trans retinoic acid: possible contributing factor in ethanol toxicity. Nutr. Rev. 68, 689–692. doi: 10.1111/j.1753-4887.2010.00323.x, PMID: 20961299

[ref59] Wozniak D. F. Hartman R. E. Boyle M. P. Vogt S. K. Brooks A. R. Tenkova T.. (2004). Apoptotic neurodegeneration induced by ethanol in neonatal mice is associated with profound learning/memory deficits in juveniles followed by progressive functional recovery in adults. Neurobiol. Dis. 17, 403–414. doi: 10.1016/j.nbd.2004.08.006, PMID: 15571976

[ref60] Xu Q. Tam M. Anderson S. A. (2008). Fate mapping Nkx2.1-lineage cells in the mouse telencephalon. J. Comp. Neurol. 506, 16–29. doi: 10.1002/cne.21529, PMID: 17990269

[ref61] Yelin R. Schyr R. B. Kot H. Zins S. Frumkin A. Pillemer G.. (2005). Ethanol exposure affects gene expression in the embryonic organizer and reduces retinoic acid levels. Dev. Biol. 279, 193–204. doi: 10.1016/j.ydbio.2004.12.014, PMID: 15708568

[ref62] Young J. K. Giesbrecht H. E. Eskin M. N. Aliani M. Suh M. (2014). Nutrition implications for fetal alcohol spectrum disorder. Adv. Nutr. 5, 675–692. doi: 10.3945/an.113.004846, PMID: 25398731 PMC4224205

[ref63] Zhang X. Subbanna S. Williams C. R. O. Canals-Baker S. Smiley J. F. Wilson D. A.. (2023). Anti-inflammatory action of BT75, a novel RARα agonist, in cultured microglia and in an experimental mouse model of Alzheimer’s disease. Neurochem. Res. 2023:3888. doi: 10.1007/s11064-023-03888-x PMC10355192 36781685

[ref64] Zhang K. Wang H. Xu M. Frank J. A. Luo J. (2018). Role of MCP-1 and CCR2 in ethanol-induced neuroinflammation and neurodegeneration in the developing brain. J. Neuroinflammation 15:197. doi: 10.1186/s12974-018-1241-2, PMID: 29976212 PMC6034273

[ref65] Zhong Y. Wu Y. Liu R. Li Z. Chen Y. Evans T.. (2011). Novel retinoic acid receptor alpha agonists for treatment of kidney disease. PLoS One 6:e27945. doi: 10.1371/journal.pone.0027945, PMID: 22125642 PMC3220717

